# Exploring Melatonin’s Multifaceted Role in Polycystic Ovary Syndrome Management: A Comprehensive Review

**DOI:** 10.7759/cureus.48929

**Published:** 2023-11-16

**Authors:** Archan Patel, Deepika Dewani, Arpita Jaiswal, Pallavi Yadav, Lucky Srivani Reddy

**Affiliations:** 1 Obstetrics and Gynaecology, Jawaharlal Nehru Medical College, Datta Meghe Institute of Higher Education and Research, Wardha, IND

**Keywords:** mood disorders, sleep disturbances, oxidative stress, hormonal imbalance, melatonin, pcos

## Abstract

Polycystic Ovary Syndrome (PCOS) is a complex endocrine disorder affecting a significant portion of the female population, characterized by hormonal imbalances, oxidative stress, sleep disturbances, and mood disorders. This review explores the multifaceted role of melatonin, a hormone primarily known for regulating circadian rhythms, in PCOS management. Melatonin's potential impact on hormonal balance, oxidative stress, sleep quality, and mood is comprehensively examined. It has been shown to enhance insulin sensitivity, regulate sex hormones, and influence gonadotropins, offering promise in addressing the intricate hormonal imbalances common in PCOS. As a potent antioxidant and anti-inflammatory agent, melatonin mitigates oxidative stress and its associated complications. Its role in improving sleep quality and mood can significantly enhance the psychological well-being and daily functioning of PCOS patients. We discuss the potential implications of melatonin as a complementary or adjunct therapy, alongside existing PCOS treatments, and its significance in improving the overall quality of life for individuals with this syndrome. While further research is needed, melatonin's multifaceted effects promise a brighter future for PCOS patients.

## Introduction and background

Polycystic Ovary Syndrome (PCOS) is a complex endocrine disorder affecting many women worldwide. Various symptoms, including irregular menstrual cycles, ovarian cysts, hyperandrogenism, and metabolic disturbances characterize it. PCOS is a leading cause of infertility and contributes to various long-term health complications, such as diabetes, cardiovascular disease, and psychological well-being [1]. PCOS affects an estimated 8-13% of reproductive-aged women. Up to 70% of affected women remain undiagnosed worldwide. Understanding and effectively managing PCOS is paramount to improving the quality of life for those affected [1].

The significance of addressing PCOS lies not only in the alleviation of its immediate symptoms but also in the prevention of associated health risks. Women with PCOS are at an increased risk of developing insulin resistance, obesity, hypertension, and dyslipidemia. Furthermore, PCOS can profoundly impact mental health, leading to anxiety, depression, and reduced quality of life. Therefore, comprehensive and tailored management strategies are essential to address both the reproductive and metabolic aspects of PCOS and the psychosocial well-being of affected individuals [2].

Melatonin, a hormone primarily known for regulating the sleep-wake cycle, has been increasingly recognized for its multifaceted properties. Beyond its circadian rhythm regulation, melatonin possesses antioxidant, anti-inflammatory, and immune-modulatory properties. Recent research suggests that melatonin may significantly impact various aspects of PCOS, including hormonal imbalances, oxidative stress, sleep disturbances, and mood disorders. This has sparked interest in exploring the potential therapeutic benefits of melatonin in managing PCOS [3].

This comprehensive review aims to provide an in-depth analysis of melatonin's multifaceted role in managing PCOS. We will examine the existing literature, encompassing preclinical and clinical studies, to elucidate the potential mechanisms through which melatonin may influence various aspects of PCOS. The review will explore melatonin's impact on hormonal balance, oxidative stress, sleep disturbances, and mood disorders in PCOS patients. By synthesizing the available evidence, we seek to shed light on the therapeutic applications of melatonin in PCOS and its implications for improving the quality of life for those affected. This review will serve as a valuable resource for researchers, healthcare providers, and individuals seeking a better understanding of the potential benefits of melatonin in PCOS management.

## Review

The role of melatonin in PCOS

Overview of Melatonin and Its Functions

Melatonin is a naturally occurring hormone primarily produced by the pineal gland in the brain, although other tissues also produce smaller amounts. Its most well-known function is the regulation of the circadian rhythm, governing the sleep-wake cycle. Melatonin levels typically rise in the evening, promoting sleep, and decline in the morning to encourage wakefulness. Beyond its role in sleep, melatonin has diverse functions in the human body [4]. These include antioxidant and free radical scavenging properties, immune system modulation, and potential effects on the endocrine system. Understanding these various roles is crucial in comprehending its potential impact on PCOS [5].

Mechanisms Underlying the Potential Benefits of Melatonin in PCOS

Regulation of circadian rhythms: As stated previously, melatonin plays a vital role in regulating circadian rhythms, which govern our sleep-wake cycles. In PCOS, where hormonal imbalances and irregular menstrual cycles are pervasive, melatonin's role in restoring regular sleep patterns is particularly significant. Disrupted sleep patterns in PCOS patients can further exacerbate hormonal imbalances. Melatonin's potential to influence the secretion of sex hormones, such as estrogen and progesterone, may help reestablish a more balanced hormonal environment. This, in turn, can contribute to managing PCOS symptoms and improve overall hormonal balance [6].

Antioxidant and anti-inflammatory effects: PCOS is characterized by elevated levels of oxidative stress and chronic inflammation, which are central to its pathophysiology. Melatonin's remarkable antioxidant properties enable it to act as a potent free radical scavenger, reducing oxidative damage to cells and tissues. Additionally, melatonin's anti-inflammatory effects help mitigate the chronic inflammation often seen in PCOS. By lowering oxidative stress and inflammation, melatonin may reduce the risk of complications associated with PCOS and enhance metabolic parameters, offering a multifaceted approach to managing the condition [7].

Impact on insulin sensitivity: Insulin resistance is a common feature of PCOS, contributing to metabolic disturbances and increasing the risk of type 2 diabetes. Research suggests that melatonin may enhance insulin sensitivity, making cells more responsive to insulin's actions. By improving insulin sensitivity, melatonin can help manage hyperinsulinemia, a common issue in PCOS, and ameliorate associated metabolic abnormalities. This effect on insulin sensitivity could have profound implications for PCOS management and reduce the long-term risk of diabetes in affected individuals [8].

Gonadotropin regulation: Melatonin receptors have been identified in the ovaries, suggesting a direct influence of melatonin on ovarian function. Melatonin may modulate the secretion of gonadotropins, such as luteinizing hormone (LH) and follicle-stimulating hormone (FSH), which are critical in regulating the menstrual cycle and ovulation. The potential for melatonin to influence the delicate balance of these hormones could have implications for restoring normal reproductive function in PCOS. By supporting ovulatory cycles, melatonin may improve fertility, a central concern for many women with PCOS. Further research is required to fully elucidate the extent of melatonin's impact on gonadotropins and its potential therapeutic application in reproductive health for PCOS patients [9].

Previous Studies and Evidence Supporting Melatonin's Role in PCOS

Hormonal balance: Melatonin is pivotal in influencing hormonal balance, especially in PCOS, where disturbances in sex hormone levels are expected. Research has provided evidence that melatonin can help restore hormonal equilibrium by reducing excess androgens, a hallmark of PCOS. Elevated androgen levels are associated with symptoms like hirsutism, acne, and irregular menstrual cycles. Melatonin's potential to lower androgen levels holds promise for alleviating these distressing symptoms and improving the overall hormonal profile in PCOS patients [10].

Oxidative stress reduction: Oxidative stress is a significant contributor to the pathophysiology of PCOS and is linked to numerous metabolic and cardiovascular risks. Melatonin's robust antioxidant properties make it an effective combatant of oxidative stress and inflammation. Studies have demonstrated that melatonin can help reduce oxidative stress and inflammation in PCOS patients, potentially ameliorating these risk factors. By mitigating oxidative stress, melatonin may protect the cardiovascular and metabolic health of individuals with PCOS, lowering the risk of diabetes and heart disease [11].

Reproductive outcomes: Melatonin's potential to improve reproductive outcomes in PCOS patients is a promising avenue of research. Some studies have explored using melatonin as an adjunct therapy to enhance menstrual regularity and promote ovulation in women with PCOS. Irregular or absent ovulation is a common issue for PCOS patients, often leading to infertility. Melatonin's influence on the menstrual cycle and ovulation may contribute to addressing this aspect of the syndrome. By restoring ovulatory cycles, melatonin could offer hope to those seeking to improve their fertility and increase the chances of conception, making it a valuable adjunct therapy in managing PCOS. Further research is needed to comprehensively understand the extent of its impact on reproductive outcomes and its potential to assist individuals with PCOS in their journey towards conception [10].

Potential Areas of Application in PCOS Management

Hormonal regulation: The potential of melatonin to restore hormonal balance is particularly relevant for individuals with PCOS experiencing androgen excess and irregular menstrual cycles. These hormonal imbalances are central to the manifestation of PCOS symptoms, including hirsutism, acne, and irregular ovulation. Melatonin's influence on sex hormones, such as estrogen, progesterone, and testosterone, holds promise for mitigating these symptoms and restoring a more balanced hormonal profile in PCOS patients. By addressing hormonal dysregulation, melatonin offers a multifaceted approach to managing the endocrine aspects of the condition [12].

Metabolic health: The potential of melatonin to improve metabolic health is of significant importance for individuals with PCOS, as the condition is often associated with insulin resistance, obesity, and metabolic disturbances. Melatonin's ability to enhance insulin sensitivity and reduce oxidative stress offers a comprehensive approach to managing the metabolic aspects of PCOS. Improved insulin sensitivity can help mitigate hyperinsulinemia and its associated risks, such as diabetes and cardiovascular complications. Melatonin's impact on metabolic parameters provides hope for long-term health benefits beyond the immediate management of PCOS symptoms [13].

Reproductive health: Melatonin's role in restoring regular ovulatory cycles and improving fertility is a critical consideration for women with PCOS who desire to conceive. Anovulation is a common issue in PCOS, leading to infertility. Melatonin's potential as an adjunct therapy to existing treatments can help promote ovulation, increasing the likelihood of successful conception. By addressing reproductive health, melatonin contributes to a more comprehensive approach to PCOS management and offers hope to those seeking to expand their families [14].

Psychosocial well-being: Beyond its effects on hormonal and metabolic parameters, melatonin's influence on sleep quality and mood is paramount. Individuals with PCOS frequently experience sleep disturbances and mood disorders, including depression and anxiety. Melatonin's ability to enhance sleep quality and mood can significantly contribute to the psychosocial well-being and overall quality of life of PCOS patients. Improved sleep quality promotes increased energy levels, concentration, and overall well-being. By addressing psychosocial well-being, melatonin offers a holistic approach to improving individuals' mental and emotional health with PCOS [15].

Melatonin's effects on hormonal imbalance in PCOS

Melatonin and Its Impact on Insulin Sensitivity

Insulin sensitizing effects: Melatonin's capacity to enhance insulin sensitivity is crucial in managing PCOS, where insulin resistance is a hallmark feature. Melatonin achieves this by potentially influencing insulin receptor signaling and improving glucose uptake in peripheral tissues. This effect holds significant clinical relevance in PCOS management, as it can help mitigate insulin resistance, which is a condition where cells do not effectively respond to insulin signals. Improved insulin sensitivity is pivotal, as it not only aids in glucose regulation but also addresses the metabolic complications often associated with PCOS, such as hyperinsulinemia and dyslipidemia. By enhancing insulin sensitivity, melatonin offers a comprehensive approach to managing the metabolic aspects of PCOS and potentially reducing the risk of type 2 diabetes [16].

Reduction in hyperinsulinemia: Hyperinsulinemia, characterized by elevated insulin levels in the bloodstream, is a common and consequential feature of PCOS. Elevated insulin levels can exacerbate the hormonal imbalances seen in PCOS, particularly by stimulating increased androgen production by the ovaries. Melatonin's role in reducing hyperinsulinemia is significant in this context. Melatonin may help lower insulin levels in individuals with PCOS by enhancing insulin sensitivity. This, in turn, can contribute to reducing androgen production, which is crucial for managing symptoms like hirsutism and acne. By addressing hyperinsulinemia, melatonin offers promise in restoring hormonal balance and alleviating the clinical manifestations of PCOS related to androgen excess [17].

Regulation of Sex Hormones (Estrogen, Progesterone, and Testosterone)

Reduction of androgens: One of the critical therapeutic aspects of melatonin in PCOS is its potential to reduce androgen levels. Both animal studies and some clinical trials support this effect. Lowering androgen levels is a crucial therapeutic goal in PCOS management, as it directly addresses the androgen excess that contributes to symptoms such as hirsutism (excessive hair growth), acne, and male-pattern hair loss. By reducing androgen levels, melatonin has the potential to alleviate these distressing androgen-related symptoms, significantly enhancing the quality of life for individuals with PCOS [18].

Influence on estrogen and progesterone: Melatonin's effects on estrogen and progesterone are multifaceted and context-dependent. While not directly acting as an estrogen or progesterone replacement, melatonin may help restore the balance between these hormones, which is particularly relevant for regulating the menstrual cycle and improving fertility in PCOS patients. Estrogen and progesterone imbalances can lead to irregular or absent menstrual cycles and difficulties in achieving pregnancy. Melatonin's role in influencing the delicate interplay between these hormones may contribute to more regular ovulatory cycles and enhanced fertility in individuals with PCOS. Further research is needed to fully elucidate the extent and specifics of melatonin's effects on estrogen and progesterone in PCOS [19].

Melatonin's Influence on Gonadotropins and Ovulation

Gonadotropin secretion: Melatonin's influence on gonadotropin secretion is a pivotal aspect of its potential role in PCOS management. Melatonin receptors have been identified in the pituitary gland, which plays a central role in regulating the secretion of LH and FSH. In PCOS, LH and FSH secretion imbalances often disrupt the normal ovulatory cycle. Melatonin may modulate the secretion of these crucial hormones, potentially restoring the regularity of the ovulatory cycle in PCOS patients. This effect holds promise for addressing one of the core issues in PCOS, as ovulatory dysfunction is a primary driver of infertility and menstrual irregularities in affected individuals [20].

Ovulation induction: Melatonin's potential to induce ovulation is particularly valuable for PCOS patients struggling with infertility due to anovulation (lack of ovulation). Some studies suggest that melatonin supplementation may contribute to improved ovulation rates in PCOS patients. This effect is of great significance, as it directly addresses one of the primary challenges faced by women with PCOS who desire to conceive. By facilitating ovulation, melatonin offers hope to those grappling with infertility, potentially increasing their chances of achieving a successful pregnancy. Further research is warranted to explore the full extent of melatonin's role in ovulation induction in PCOS and its implications for improving fertility in affected individuals. Table 1 describes the effect of melatonin on PCOS [21].

**Table 1:** Effects of melatonin in PCOS PCOS: Polycystic Ovary Syndrome

Effects of Melatonin in PCOS	Description
Hormonal Balance	Potential reduction in androgen levels, addressing hirsutism, acne, and male-pattern hair loss. Influence on estrogen and progesterone balance, promoting regular menstrual cycles and improving fertility.
Metabolic Health	Enhancement of insulin sensitivity, aiding in glucose regulation and reducing the risk of type 2 diabetes. Reduction in hyperinsulinemia, mitigating insulin-driven androgen production and addressing symptoms associated with androgen excess. Management of metabolic complications, such as hyperinsulinemia and dyslipidemia.
Reproductive Health	Potential ovulation induction, addressing infertility related to anovulation. Improved egg quality and embryo development, increasing the chances of successful pregnancy. Restoration of ovulatory cycles, enhancing fertility in individuals with PCOS.
Psychosocial Well-being	Enhanced quality of sleep, leading to increased energy, better concentration, and improved overall well-being. Alleviation of mood disorders, such as depression and anxiety, contributing to better mental health, improved relationships, and increased life satisfaction.
Mitigation of Metabolic Abnormalities	Reduction in oxidative stress, potentially lowering the risk of chronic health conditions like cardiovascular disease and diabetes.
Alleviation of Androgen Excess	Reduction of androgen levels, addressing symptoms like hirsutism, acne, and male-pattern hair loss.
Improvement in Reproductive Outcomes	Restoration of regular ovulatory cycles, crucial for women with PCOS seeking fertility. Enhanced fertility due to improved egg quality and embryo development.
Reduction of Cardiovascular Risk	Lowering oxidative stress and improving insulin sensitivity may reduce the long-term cardiovascular risk associated with PCOS.
Sleep-Wake Cycle Regulation	Promotion of regular sleep patterns, improving overall sleep quality. Reduction in time to fall asleep and enhancement of sleep maintenance, potentially alleviating insomnia and other sleep-related issues.
Mood Enhancement	Positive impact on mood due to improved sleep quality. Reduction in the risk of depression and anxiety, enhancing emotional well-being.

Melatonin and oxidative stress in PCOS

The Relationship between Oxidative Stress and PCOS

Increased reactive oxygen species (ROS) production: Oxidative stress is a pivotal factor in the pathophysiology of PCOS. It is characterized by an imbalance between ROS production and the body's ability to neutralize them with antioxidants. In PCOS, ROS is a notable overproduction, including free radicals like superoxide anion and hydrogen peroxide. These excess ROS can cause damage to cellular structures, leading to oxidative damage and inflammation. Importantly, this excessive ROS production is intricately linked to several PCOS-related complications, including insulin resistance, hyperandrogenism (elevated levels of male hormones), and endothelial dysfunction. The connection between ROS and these complications underscores the significance of oxidative stress in PCOS pathophysiology [22].

Antioxidant defense mechanisms: An integral component of the oxidative stress imbalance in PCOS is the reduced activity of antioxidant defense mechanisms. PCOS patients often exhibit decreased levels and activity of endogenous antioxidants. This reduction in antioxidant capacity leaves the body less equipped to counteract the damaging effects of ROS. Consequently, the imbalance between ROS production and the body's capacity to neutralize them with antioxidants exacerbates oxidative stress in PCOS. Addressing this antioxidant deficit is a critical consideration in managing the condition, as it may help mitigate the complications associated with PCOS, such as insulin resistance and the hyperandrogenic state [23].

Melatonin's Role as an Antioxidant and Its Effects on Oxidative Stress

ROS scavenging: Melatonin can be a direct scavenger of ROS and reactive nitrogen species (RNS). Melatonin effectively reduces oxidative damage to cells and tissues by neutralizing these harmful free radicals. This antioxidative property is paramount in PCOS, where an overproduction of ROS contributes to the condition's pathophysiology. By reducing the burden of oxidative stress, melatonin can help mitigate the damage associated with PCOS, potentially alleviating complications such as insulin resistance, hyperandrogenism, and endothelial dysfunction [24].

Enhancement of endogenous antioxidant defenses: Melatonin goes beyond ROS scavenging by supporting and enhancing the activity of endogenous antioxidant enzymes, including superoxide dismutase (SOD) and glutathione peroxidase (GPx). These enzymes play a pivotal role in the body's defense against oxidative stress, helping to neutralize free radicals and maintain cellular integrity. By strengthening the activity of these endogenous antioxidants, melatonin further bolsters the body's capacity to combat oxidative stress. This effect is of considerable importance in managing PCOS, as it addresses the deficit in antioxidant defenses often observed in affected individuals [25].

Anti-inflammatory effects: Inflammation and oxidative stress are closely intertwined processes, often exacerbating each other. Melatonin's anti-inflammatory properties are precious in PCOS, as the condition is associated with increased production of pro-inflammatory cytokines. By reducing the production of these inflammatory molecules, melatonin indirectly lowers oxidative stress. This interplay between inflammation and oxidative stress is integral to the pathophysiology of PCOS, and by reducing inflammation, melatonin contributes to a comprehensive approach to mitigating the oxidative stress burden in PCOS. This, in turn, may help address the metabolic and reproductive complications associated with the condition [26].

Implications for PCOS Management

Mitigation of metabolic abnormalities: Melatonin's multifaceted effects, including its role in reducing oxidative stress and improving insulin sensitivity, are significant in addressing the metabolic abnormalities frequently observed in individuals with PCOS. By mitigating oxidative stress, melatonin may reduce metabolic complications, such as hyperinsulinemia (elevated insulin levels), obesity, and dyslipidemia. Improved insulin sensitivity is pivotal in regulating glucose metabolism and preventing the onset of type 2 diabetes. Melatonin's potential to enhance metabolic health offers a comprehensive approach to managing the metabolic aspects of PCOS, ultimately reducing the risk of diabetes and cardiovascular complications [27].

Alleviation of androgen excess: Melatonin's antioxidative properties have the potential to contribute to the reduction of androgen levels in individuals with PCOS. Elevated androgens are central to presenting androgen-related symptoms in PCOS, including hirsutism (excessive hair growth) and acne. By lowering androgen levels, melatonin may help manage these distressing symptoms, improving the quality of life for PCOS patients. The alleviation of androgen excess is a critical therapeutic goal in PCOS management, and melatonin's influence on this aspect offers hope for individuals affected by the condition [28].

Improvement in reproductive outcomes: Lowering oxidative stress is intricately linked to restoring regular ovulation, a primary concern for women with PCOS who desire fertility. Melatonin's potential impact on reproductive health extends beyond restoring ovulation to potentially improving egg quality and embryo development. By enhancing the reproductive aspects of PCOS, melatonin may offer hope to those seeking to conceive and increase their chances of successful pregnancies. This effect underscores the importance of melatonin as an adjunct therapy for PCOS, addressing not only the metabolic but also the reproductive aspects of the condition [29].

Reduction of cardiovascular risk: Oxidative stress is a known risk factor for cardiovascular disease. Lowering oxidative stress through melatonin supplementation may contribute to the reduction of long-term cardiovascular risk in individuals with PCOS. The condition is associated with an increased risk of heart disease, and by addressing oxidative stress, melatonin potentially plays a role in mitigating this risk. By reducing cardiovascular risk factors, melatonin offers a broader health benefit beyond the immediate management of PCOS symptoms, highlighting its significance as a potential therapeutic intervention for this complex syndrome [30].

Melatonin's impact on sleep and mood disorders in PCOS

Sleep Disturbances and Mood Disorders in PCOS

Sleep disturbances: Sleep disturbances are prevalent among women with PCOS. These disturbances can take various forms, including insomnia, sleep apnea, and restless leg syndrome. The link between PCOS and sleep disturbances is complex and multifactorial. Hormonal imbalances, such as elevated androgens and insulin resistance, may contribute to sleep disruption in PCOS patients. Additionally, obesity, a common comorbidity in PCOS, can exacerbate sleep apnea and other sleep-related issues. Sleep disturbances can significantly impact the quality of life and overall well-being of individuals with PCOS, leading to daytime fatigue, impaired concentration, and decreased productivity. Addressing these sleep issues is crucial in improving the psychological and physical health of PCOS patients [15].

Mood disorders: Mood disorders, including depression and anxiety, are more prevalent in individuals with PCOS than in the general population. These conditions can profoundly impact a person's quality of life and overall well-being. The exact mechanisms linking PCOS and mood disorders are complex and poorly understood. Hormonal imbalances, such as elevated androgens and irregular menstrual cycles, may play a role in developing mood disorders. The distressing physical symptoms associated with PCOS, like hirsutism and acne, can also contribute to a negative body image and psychological distress. The challenges associated with fertility and managing PCOS-related symptoms can also contribute to heightened stress and emotional struggles. Addressing mood disorders is critical to PCOS management, as improving mental well-being is integral to enhancing the overall quality of life for individuals living with the condition [31].

Melatonin's Role in Improving Sleep Quality and Mood

Sleep-wake cycle regulation: Melatonin supplementation can regulate the sleep-wake cycle, which is often disrupted in individuals with PCOS. Irregular sleep patterns are a common issue in PCOS and can further exacerbate symptoms. By promoting a more regular sleep pattern, melatonin can help individuals with PCOS achieve a more balanced circadian rhythm. This, in turn, contributes to an improvement in the overall quality of sleep, leading to increased energy levels, enhanced cognitive function, and a greater sense of well-being [32].

Sleep onset and maintenance: Melatonin's effects extend to reducing the time it takes to fall asleep and improving sleep maintenance. This potential alleviation of insomnia and other sleep-related issues is significant to PCOS patients who frequently experience sleep onset and maintenance difficulties. By shortening the time to fall asleep and promoting uninterrupted sleep, melatonin can enhance the quantity and quality of rest. This translates to reduced daytime fatigue and improved mental and physical health for individuals with PCOS [33].

Mood enhancement: Melatonin's influence on the sleep-wake cycle is inherently linked to mood enhancement. Improved sleep quality, resulting from a more regular sleep pattern, can positively impact mood. Individuals with PCOS are more susceptible to mood disorders, including depression and anxiety. Addressing sleep disturbances is a crucial aspect of managing these mood disorders. By enhancing the emotional well-being of PCOS patients through improved sleep quality, melatonin can reduce the risk of mood disorders, contributing to an overall enhancement in the mental health and quality of life of individuals living with the condition [34].

Implications for Overall Quality of Life in PCOS Patients

Enhanced quality of sleep: One of the critical benefits of melatonin in PCOS management is the potential to improve sleep quality. Better sleep quality leads to increased energy levels, improved concentration, and an overall sense of well-being. For individuals with PCOS, this means better daily functioning and increased productivity. Enhanced sleep quality is integral to addressing the daytime fatigue often experienced by PCOS patients, allowing them to engage more effectively in their daily activities and tasks [35].

Psychological well-being: Melatonin's role in alleviating mood disorders, such as depression and anxiety, profoundly enhances the psychological well-being of individuals with PCOS. Improved mental health can positively impact relationships, self-esteem, and overall life satisfaction. PCOS can be emotionally challenging due to its physical symptoms and fertility concerns. Addressing mood disorders is essential for reducing emotional distress and improving the overall quality of life for PCOS patients [36].

Fertility and reproductive outcomes: Improved sleep quality and mood can indirectly contribute to the success of fertility treatments and increase the chances of conceiving, which is often a significant concern for women with PCOS. Fertility treatments are more likely to be effective when the body is well-rested and stress is reduced. By enhancing sleep and mood, melatonin can improve reproductive outcomes, providing hope to those seeking to start or expand their families [37].

Long-term health: The benefits of addressing sleep disturbances and mood disorders in PCOS extend beyond short-term well-being and have implications for long-term health. Reducing stress and improving sleep quality can lower the risk of chronic health conditions, such as cardiovascular disease and diabetes, associated with PCOS. By improving sleep and mood, melatonin may contribute to a broader health benefit, reducing the risk of long-term complications and improving the overall health and well-being of individuals with PCOS [15].

Potential therapeutic applications of melatonin in PCOS

Melatonin Supplementation and Dosages

Dosage and timing: Establishing the correct dosage and timing of melatonin supplementation is a crucial aspect of using melatonin in PCOS management. The appropriate dosage can vary among individuals and should be tailored to the patient's needs. Factors to consider in determining the correct dosage may include the individual's age, weight, and the severity of PCOS symptoms. Clinical trials and research studies can provide valuable insights into optimal dosing regimens, helping healthcare providers make informed recommendations for their patients. Close monitoring of the patient's response and potential side effects is also essential when determining the ideal dosage and timing for melatonin supplementation [38].

Long-term vs. short-term use: The decision regarding the duration of melatonin use in PCOS should be carefully considered. It's essential to evaluate whether melatonin is being used as a short-term intervention to address specific PCOS-related issues, such as sleep disturbances and mood disorders, or as a long-term therapy to maintain hormonal balance, improve sleep quality, and manage oxidative stress. The choice between short-term and long-term use should consider the patient's needs and the specific therapy goals. Monitoring the patient's progress and assessing the ongoing benefits and potential side effects of melatonin supplementation will help guide decisions regarding the duration of use. The ultimate aim is to balance optimizing PCOS management and ensuring patient safety and well-being [10].

Combining Melatonin with Existing PCOS Treatments

Adjunct therapy: Melatonin can be an effective adjunct therapy alongside conventional PCOS treatments, including oral contraceptives, insulin-sensitizing medications, and lifestyle modifications. This combined approach may offer more comprehensive results by simultaneously addressing multiple facets of PCOS. While conventional treatments focus on hormonal and metabolic aspects of the condition, melatonin complements the management of sleep disturbances, mood disorders, and oxidative stress. The synergy of these treatments can result in a more well-rounded approach to PCOS management, potentially leading to improved patient outcomes and quality of life [10].

Personalized treatment plans: Developing personalized treatment plans tailored to each PCOS patient's specific needs and symptoms is crucial. When recommending combined therapies, healthcare providers should consider the individual's clinical profile, including hormonal imbalances, metabolic status, and sleep disturbances or mood disorders. Healthcare providers can better address each patient's unique challenges and goals by taking a personalized approach. This tailored strategy optimizes treatment regimens, ensuring that patients receive the most practical combination of therapies to manage their PCOS symptoms. Personalized treatment plans consider the multifaceted nature of PCOS and acknowledge that no single approach is suitable for every patient [39].

Safety and Potential Side Effects

Safety profile: Melatonin is generally considered safe when used as directed. However, healthcare providers need to discuss potential side effects with patients. Common side effects may include drowsiness, nausea, and headache. Patients should be informed about these potential side effects, and healthcare providers should monitor their tolerance to melatonin, especially when initiating treatment. It is also vital to emphasize the importance of using melatonin by recommended dosages and timing to minimize the risk of adverse effects [40].

Interactions with medications: Healthcare providers must be vigilant regarding potential drug interactions when combining melatonin with other PCOS treatments or medications. Interactions can affect the efficacy and safety of melatonin and concomitant drugs. Careful consideration of the patient's medication regimen is essential to identify and manage potential interactions. Healthcare providers should be prepared to adjust treatment plans to optimize therapy while minimizing the risk of adverse interactions. Table 2 mentions the potential side effects of the melatonin [41]. Table 3 mentions the potential drug interactions with melatonin in PCOS [41].

**Table 2:** Potential Side Effects of Melatonin in PCOS PCOS: Polycystic Ovary Syndrome

Potential Side Effects	Description
Drowsiness	Melatonin can cause drowsiness, especially when taken in higher doses or at inappropriate times. Patients should be advised not to operate heavy machinery or engage in activities requiring full alertness after melatonin ingestion.
Nausea	Some individuals may experience nausea as a side effect of melatonin supplementation. This should be discussed with patients to gauge their tolerance and adjust dosages if necessary.
Headache	Headaches may occur as a side effect of melatonin use. Patients should be made aware of this potential effect and instructed on managing any discomfort.
Gastrointestinal Disturbances	Melatonin can occasionally lead to gastrointestinal disturbances, such as stomach discomfort or diarrhea. Patients should report any such symptoms to their healthcare providers for evaluation and potential adjustments to their melatonin regimen.

**Table 3:** Potential Drug Interactions with Melatonin in PCOS PCOS: Polycystic Ovary Syndrome

Drug Interactions	Description
Sedatives and Hypnotics	Melatonin may enhance the effects of sedative and hypnotic medications, potentially leading to increased drowsiness and cognitive impairment. - Patients should be cautious when combining melatonin with these drugs and should consult their healthcare providers for guidance.
Anticoagulants	There is a theoretical risk of increased bleeding when melatonin is taken alongside anticoagulant medications. - Patients using anticoagulants should discuss the potential interactions with their healthcare providers.
Immunosuppressants	The immune-enhancing effects of melatonin may interact with immunosuppressant drugs, potentially affecting their efficacy. - Patients taking immunosuppressants should consult with their healthcare providers before using melatonin.
Antihypertensive Medications	Melatonin may alter blood pressure, potentially affecting the efficacy of antihypertensive drugs. - Patients on antihypertensive medications should discuss melatonin supplementation with their healthcare providers.
Anticonvulsants	Melatonin may influence the efficacy of anticonvulsant medications, potentially affecting seizure control. - Patients taking anticonvulsants should consult with their healthcare providers before using melatonin.

Pregnancy and lactation: Special consideration should be given to the safety of melatonin during pregnancy and breastfeeding, as data in these populations are limited. Healthcare providers should exercise caution when recommending melatonin to pregnant or lactating individuals with PCOS. The potential benefits of melatonin must be weighed against the potential risks, considering the individual's specific clinical profile and the status of their pregnancy or breastfeeding. In such cases, engaging in thorough discussions with patients is essential, providing them with informed choices and considering alternative therapies as necessary to ensure the well-being of both the mother and the child. Patient safety and optimal care should be the guiding principles in these situations [42].

Future Research Directions

Clinical trials: Conducting well-designed clinical trials is imperative to establish the efficacy and safety of melatonin in PCOS management. These trials should rigorously examine various aspects, including its impact on hormonal balance, oxidative stress, sleep quality, mood, and the overall quality of life in PCOS patients. Controlled and well-powered clinical trials can provide concrete evidence of melatonin's benefits and guide healthcare providers in recommending it as a therapeutic option for PCOS [43].

Mechanistic studies: In-depth mechanistic studies are essential to further our understanding of how melatonin influences PCOS-related factors. This includes investigating its effects on insulin sensitivity, regulation of sex hormones, and gonadotropin secretion. A comprehensive understanding of these mechanisms can help fine-tune melatonin treatment strategies and enhance its effectiveness in addressing the specific aspects of PCOS [43].

Long-term outcomes: Exploring the long-term effects of melatonin supplementation is vital. This includes assessing its impact on fertility, cardiovascular health, and metabolic parameters over extended periods. Longitudinal studies can provide valuable insights into the sustained benefits of melatonin in PCOS and help assess its role in preventing or mitigating long-term complications associated with the condition [44].

Individualized approaches: Research into developing individualized treatment plans is essential. These plans should consider patient-specific factors, including genetic variations, to optimize the therapeutic benefits of melatonin in PCOS. Personalized treatment approaches can enhance the precision and efficacy of melatonin therapy, ensuring that each patient receives the most tailored and effective treatment for their unique clinical profile. By incorporating individualized approaches, healthcare providers can maximize the potential benefits of melatonin while minimizing potential risks and side effects for PCOS patients. Table 4 describe the potential therapeutic applications of melatonin in PCOS [45].

**Table 4:** Potential Therapeutic Applications of Melatonin in PCOS PCOS: Polycystic Ovary Syndrome

Potential Therapeutic Applications of Melatonin in PCOS	Description
Hormonal Regulation	Reduction of androgen levels, alleviating symptoms such as hirsutism, acne, and male-pattern hair loss. Influence on estrogen and progesterone balance, promoting regular menstrual cycles and enhancing fertility.
Metabolic Management	Enhancement of insulin sensitivity, aiding in the regulation of glucose levels and mitigating the risk of type 2 diabetes. Reduction of hyperinsulinemia, addressing insulin-driven androgen production and symptoms associated with androgen excess. Management of metabolic complications, such as hyperinsulinemia and dyslipidemia, contributing to overall metabolic health improvement.
Reproductive Support	Facilitation of ovulation, potentially addressing infertility related to anovulation. Improvement in egg quality and embryo development, enhancing the chances of successful pregnancy. Restoration of ovulatory cycles, fostering fertility in individuals with PCOS.
Psychosocial Well-Being	Promotion of better sleep quality, resulting in increased energy, improved concentration, and overall well-being. Alleviation of mood disorders, including depression and anxiety, leading to better emotional health, improved interpersonal relationships, and enhanced life satisfaction.
Mitigation of Metabolic Abnormalities	Reduction of oxidative stress, potentially lowering the risk of chronic conditions such as cardiovascular disease and diabetes.
Improvement in Cardiovascular Health	Lowering oxidative stress and enhancing insulin sensitivity, possibly reducing the long-term cardiovascular risks associated with PCOS.

## Conclusions

In conclusion, melatonin's multifaceted role in the management of PCOS offers a promising avenue for addressing the complexities of this condition. Melatonin's ability to influence hormonal balance, mitigate oxidative stress, improve sleep quality, and enhance mood has the potential to impact the lives of PCOS patients significantly. While it may not replace established treatments, its complementary or adjunct therapy role is paramount. Melatonin's relatively safe profile and minimal side effects make it an attractive option for many PCOS patients. By enhancing the overall quality of life through the restoration of regular menstrual cycles, reduced hyperandrogenism, improved insulin sensitivity, and fertility enhancement, melatonin offers hope for a brighter future for those affected by this challenging syndrome. As research in this field continues to evolve, the multifaceted effects of melatonin remain an area of active investigation, holding the promise of improving the lives of individuals living with PCOS.
